# Dynamic change in maternal cardiac function during pregnancy

**DOI:** 10.3389/fcvm.2025.1577213

**Published:** 2025-06-06

**Authors:** Xiu-Juan Wang, Ling-Ling Chen, Ming-Huan Hong, Ling-Yun Kong, Wei Xiang, Li Fu, Xiao-Wei Li, Fang Liu

**Affiliations:** Department of Cardiovascular Disease, Beijing Tsinghua Changgung Hospital, School of Clinical Medicine, Tsinghua Medicine, Tsinghua University, Beijing, China

**Keywords:** cardiac function, remodeling, pregnancy, speckle-tracking echocardiography, tissue doppler imaging

## Abstract

**Background:**

Pregnant women experience various physiological changes that, if uncompensated, may result in varying degrees of cardiac dysfunction, and adverse pregnancy outcomes. Left ventricular (LV) global longitudinal strain (GLS) and P-wave to A' duration on tissue Doppler imaging (PA-TDI) have been shown to be able to detect subtle cardiac dysfunction.

**Methods:**

The present study was a prospective cross-sectional study. A total of 506 healthy pregnant women were enrolled, including 149 during early pregnancy (before 13 weeks' gestation, T1 group), 99 during mid-pregnancy (14–27 weeks' gestation, T2 group), and 258 during late pregnancy (after 28 weeks' gestation, T3 group), while 172 age- and baseline weight-matched healthy nonpregnant women served as the control group (NPC group). Clinical and echocardiographic data of the subjects were collected. The difference in cardiac structure and function among the 4 groups were analyzed. Multivariate regression analysis was conducted to identify the independent factors influencing change in cardiac function.

**Results:**

The median age of the 4 groups were comparable [T1 group, 31.0 (28.5,34.0) years; T2 group, 31.0 (29.0,34.0) years; T3 group, 31.0 (29.0,34.0) years; the NPC group, 31.0 (28.0,34.0) years, *P* = 0.905). Left ventricular ejection fraction (LVEF) during late pregnancy was lower than that during early pregnancy and the control group, but remained within normal range. With the increase of gestational age, the absolute value of LV-GLS decreased gradually [T1 group, −19.00 (−21.40, −16.70); T2 group, −17.40 (−20.10, −15.30); T3 group, −16.35 (−17.93, −13.97); *P* < 0.001]. PA-TDI during the third trimester was longer than that in the first [117.65 (108.45,128.03) ms vs. 114.19 (105.61,121.11) ms, *P* = 0.012] or the second trimester [111.32 (107.27,121.11) ms, *P* = 0.010]]. Multivariate regression analysis showed that gestational age was independently associated with LV-GLS (*b* = 0.096, *t* = 2.212, *P* = 0.027) and PA-TDI (*b* = 0.158, *t* = 2.449, *P* = 0.014).

**Conclusion:**

Pregnant women show a trend toward decreased left ventricular systolic and diastolic function. PA-TDI and LV-GLS can be used to evaluate subtle change in left cardiac function in pregnant women.

## Introduction

Cardiovascular diseases are the leading cause of maternal mortality (1), accounting for over 33% of pregnancy-related death (2). Previous study about the change of cardiac systolic and diastolic function during pregnancy has been controversial. Some studies found a declining trend in cardiac function during pregnancy, but previous research focused primarily on the middle and late trimesters, with a lack of assessment during the early trimester and a relatively small sample size (3–10).

Echocardiography is the primary imaging modality for evaluation of the cardiac function in pregnant women. Global longitudinal strain (GLS) has been well validated to detect subtle myocardial dysfunction (11, 12). Recently, P-wave to A' duration on tissue Doppler imaging (PA-TDI), a novel parameter based on electrical and echocardiographic data, has been shown to be more sensitive and reliable in assessing left ventricular diastolic function in some population (13, 14)^.^ PA-TDI duration is calculated as the time difference between the start of the P-wave on the surface electrocardiogram and the peak of the A'-wave on the TDI tracing (15). In addition, the PA-TDI duration can be measured immediately on the echo machine without the need for time-consuming offline post-processing and has minimal intra- and interobserver variability (13). As yet, PA-TDI has not been applied in the pregnant population. Therefore, the aim of our study was to assess early change in systolic and diastolic function in women during pregnancy with GLS and PA-TDI.

## Methods

### Study population and protocol

This was a cross-sectional observational study conducted at Beijing Tsinghua Changgung Hospital, between November 2021 and August 2024. The participants was administered echocardiography examination at the discretion of the clinician. Inclusion criteria were as follows: Women aged 20 to 45 years who are pregnant during the early (0–13 weeks, T1 group), middle (14–27 weeks, T2 group), and late (28 weeks to delivery, T3 group) trimesters. The exclusion criteria included those with hypertension, diabetes, stroke, and structural heart disease (such as congenital heart disease, valvular heart disease, and cardiomyopathy); persistent arrhythmias, immune system diseases, and those with serious respiratory diseases. The following demographic characteristics were collected: age, height, weight, body mass index (BMI), body surface area (BSA), blood pressure, heart rate. The baseline weight before pregnancy of the pregnant women was recorded. Age- and baseline weight-matched healthy non-pregnant women were included as the control group (NPC group). The study was approved by the Beijing Tsinghua Changgung hospital's Ethics Committee (22021-4-05). All participants provided written informed consent.

### Transthoracic echocardiography

The Vivid E9 or Vivid E95 (GE Healthcare) ultrasound system were used, with probe frequency ranging from 3.5 to 5.5 MHz. Participants were instructed to lie in the left lateral decubitus position and breathe calmly, while electrocardiogram were recorded synchronously. After routine transthoracic echocardiography using the M5S probe, the probe was switched to the 4 V probe. Tri-plane mode was activated to display the apical 4-, 2-, and 3 chamber views simultaneously (frame rate: 40–60 frames/sec).

According to the American Society of Echocardiography (ASE) guidelines, the following echocardiographic parameters were measured. The left atrial (LA) anterioposterior diameter (LAD) was measured perpendicular to the long axis of the atrium at the parasternal long axis view. Left ventricular (LV) internal diameter at end-systole (LVIDs) and end-diastole (LVIDd), along with interventricular septal thickness (IVST) and posterior wall thickness (PWd) were measured in the M mode. LV mass (LVM, in grams) was derived using the Devereux formula: LVM = 0.8 × (1.04 × (LVIDd + PWd + IVST)^−^3—LVIDd^−^3) + 0.6. LVM was normalized to BSA to calculate the LV mass index (LVMI). Relative wall thickness (RWT) was determined as: RWT = 2 × PWd/LVIDd. LV end-diastolic (EDV), end-systolic volume (ESV), and ejection fraction (LVEF) were calculated using the Biplane Simpson's method and stroke volume (SV) and stroke index (SI) were derived.

Pulsed-wave Doppler sampling was placed at the level of the mitral valve tips to obtain peak early diastolic (E wave) and late diastolic (A wave) flow velocities, and the E/A ratio was calculated. With tissue Doppler imaging, the sample volume was placed at the basal interventricular septal and the basal left ventricular lateral wall to get the average of peak early diastolic myocardial motion velocity (e'), and the E/e' ratio was calculated.

**Figure 1:**
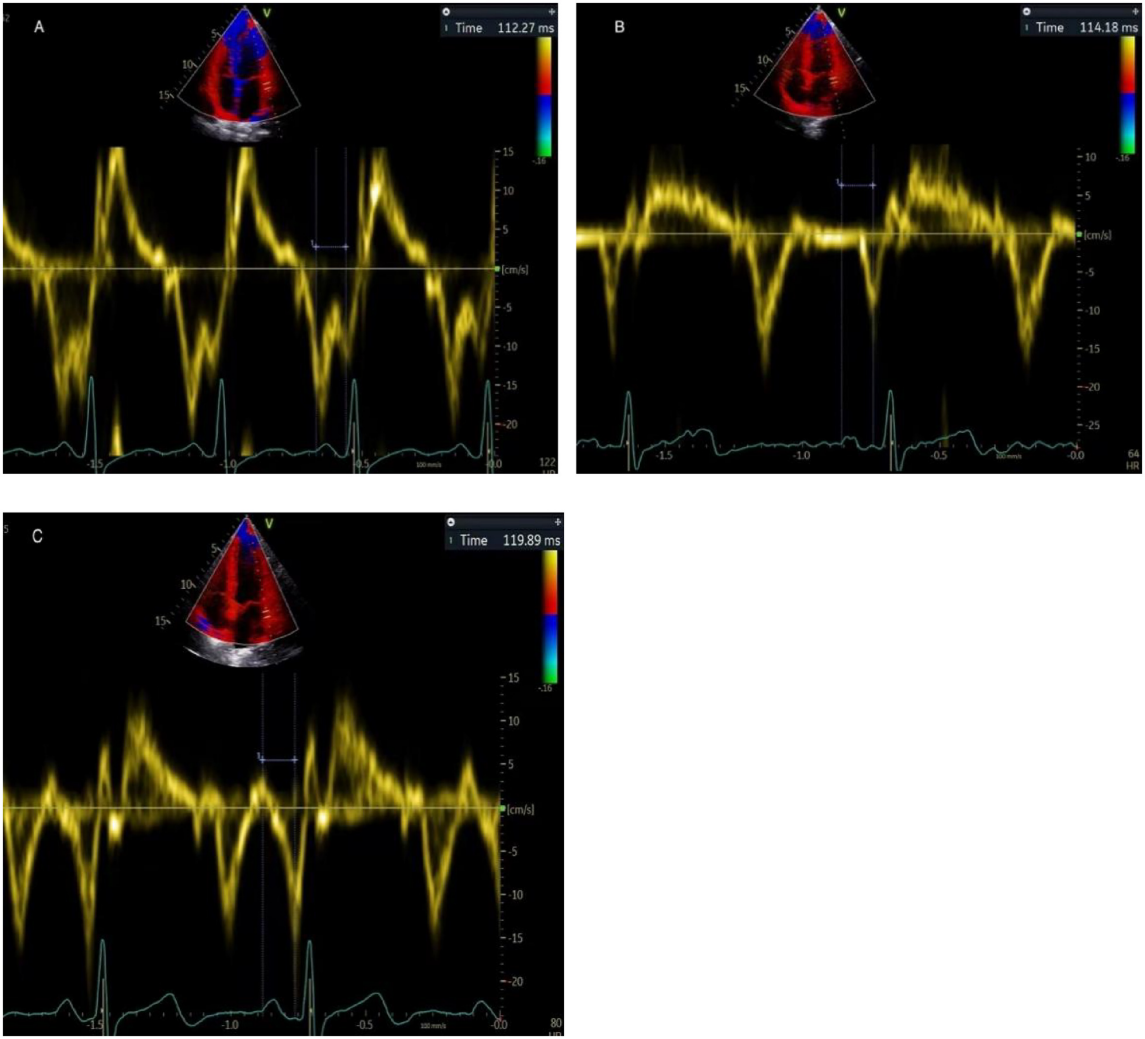
Depiction of measuring PA-TDI duration in the same patient. It is calculated using the interval between the beginning of the P-wave on the surface electrocardiogram and the peak of the A'-wave on the TDI tracing. **(A)** early pregnancy, **(B)** middle pregnancy, **(C)** late pregnancy.

The PA-TDI duration was calculated using color-coded TDI imaging in the apical 4-chamber view (Figure 1). A fixed 8 mm × 8 mm region of interest was positioned on the lateral LA wall, immediately above the mitral annulus to trace mechanical activation of this region. The time interval from the beginning of the P-wave on the surface electrocardiogram to the peak of the A'-wave on the TDI tracing was used to measure the PA-TDI duration (14).

**Figure 2:**
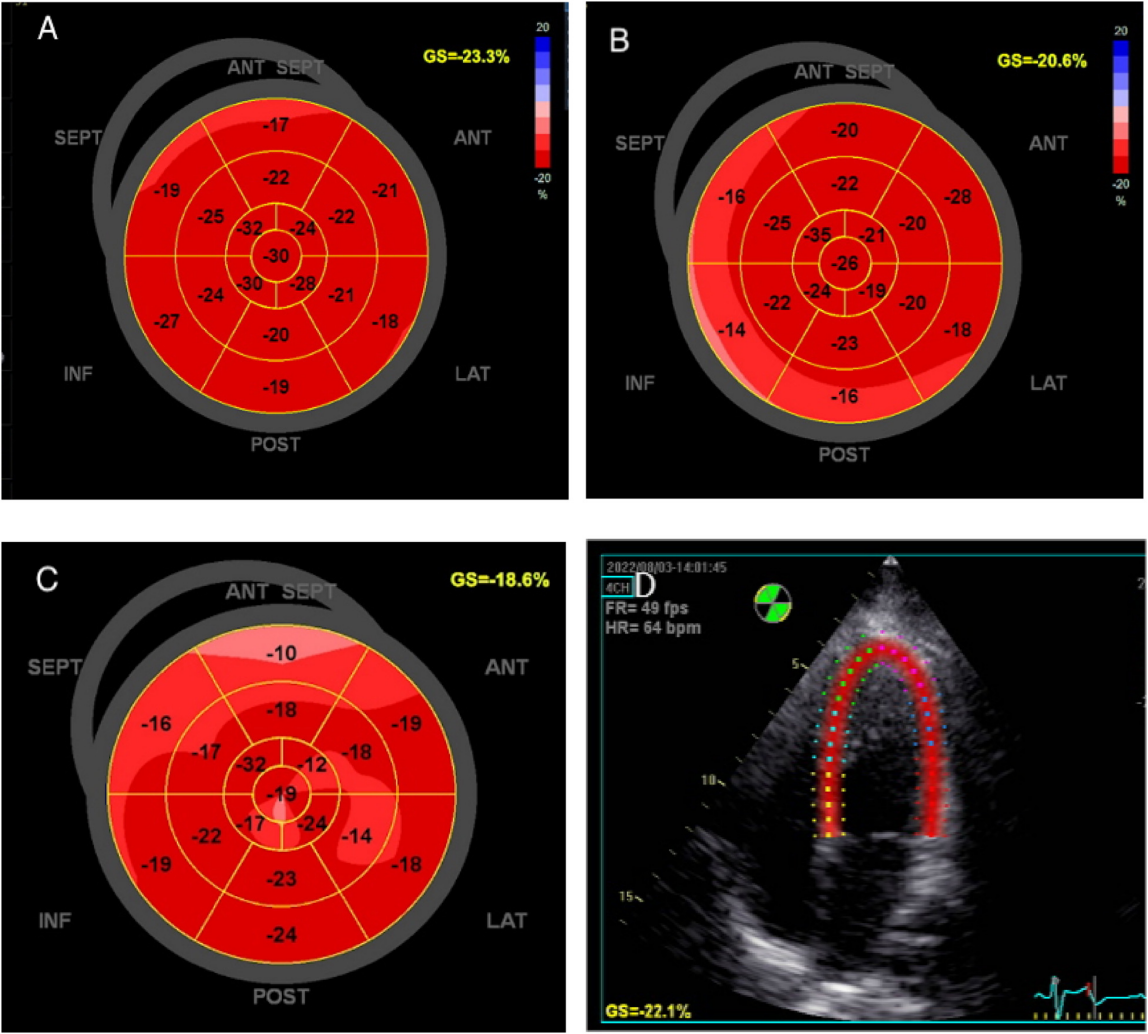
The global longitudinal strain of left ventricle was measured by speckle tracking imaging in the same patient. **(A)** early pregnancy, **(B)** middle pregnancy, **(C)** late pregnancy, **(D)** Speckle tracking imaging.

Two-dimensional speckle tracking imaging was used to measure LV GLS. The endocardial and epicardial borders of LV were automatically traced, with appropriate adjustments made to ensure the entire LV wall was encapsulated. LV GLS was then automatically calculated (Figure 2). All the images were acquired by two experienced professional physicians, and another physician, who was blinded to the baseline clinical data, performed the analysis. All parameters were measured for three times and the average value was used for analysis.

### Statistical analysis

Continuous variables are described as mean ± SD or median and interquartile range (IQR). The normality of distribution was tested using the Shapiro Wilk test. Differences among the four groups were analyzed using one-way ANOVA and the Kruskal-Wails H test, and differences between two groups were tested using the Student's *t* test or the Mann–Whitney *U*-test. Multiple linear regression analysis was used to determine the independent influencing factors of echocardiographic parameters. *P* value < 0.05 was considered statistically significant. The analysis was performed with SPSS version 26 software (IBM Corp., Armonk, NY, USA).

## Results

### Clinical characteristics

A total of 506 healthy pregnant women were prospectively enrolled, including 149 cases in the first, 99 in the second, and 258 cases in the third trimesters. Also included were 172 non-pregnant women matched in terms of age and baseline weight (Table 1). The median week of gestation were 9 w during early, 20 w during mid, and 36 w during late gestation, respectively. The median age was 31 years among the three trimesters [T1 group, 31.0 (28.5, 34.0); T2 group, 31.0 (29.0, 34.0); T3 group, 31.0 (28.0, 34.0); *P* > 0.05]. Three participants underwent sequential echocardiographic examinations across the first, second, and third trimesters of pregnancy. There was no statistically significant difference in age, height, systolic blood pressure, and baseline height among the four groups (*P* > 0.05). However, The weight, BMI, and BSA of late-pregnant women were higher than those of early- and mid-pregnant women and the control group (*P* < 0.05). The diastolic blood pressure in the second trimester was lower than in the third trimester (*P* = 0.001) and the control group (*P* = 0.035). The heart rate in the second and third trimesters was faster than in the first trimester and the control group (*P* < 0.05).

**Table 1:** Clinical characteristics of the healthy pregnancy group and control group.

Characteristic	T1 (*n* = 149)	T2 (*n* = 99)	T3 (*n* = 258)	NPC (*n* = 172)	*P*
Age (years)	31.0 (28.5,34.0)	31.0 (29.0,34.0)	31.0 (29.0,34.0)	31.0 (28.0,34.0)	0.905
Height(m)	1.63 (1.60,1.66)	1.58 (1.63,1.66)	1.63 (1.60,1.65)	1.63 (1.60,1.67)	0.947
Pre-pregnancy weight (kg)	57.0 (51.0,64.0)	57.0 (52.0,62.0)	55.0 (51.0,62.0)	59.0 (53.0,65.0)	0.051
Weight (kg)	58.0 (52.0,63.5)^§^	60.0 (55.0,69.0)^¶^	68.0 (62.6,74.5)	59.0 (53.0,65.0)	<0.001
BMI	21.67 (19.94,23.51)^§^	22.86 (21.5,25.24)^¶^	25.70 (23.78,27.59)	22.05 (20.20,24.28)	<0.001
BSA	1.62 (1.52,1.72)^§^	1.62 (1.52,1.72)^¶^	1.73 (1.66,1.73)	1.63 (1.55,1.71)	<0.001
SBP (mmHg)	111 (105,120)	103 (108,117)	111 (103,118)	110 (104,117)	0.226
DBP (mmHg)	69 (64,76)	67 (63,75)^†^^,^^¶^	72 (67,76)	71 (65,77)	0.001
HR (bpm)	77 (67,85)^$^^,^^§^	83 (75,93)^†^	86 (78,95)*	73 (68,79)	<0.001

Data are expressed as mean ± standard deviation or median (interquartile range). BMI, body mass index; BSA, body surface area; SBP, systolic pressure; DBP, diastolic blood pressure; HR, heart rate.

* *P* < 0.05, T3 vs. NPC.

^†^ *P* < 0.05, T2 vs. NPC.

^‡^ *P* < 0.05, T1 vs. NPC.

^$^ *P* < 0.05, T1 vs. T2.

^§^ *P* < 0.05, T1 vs. T3.

^¶^ *P* < 0.05, T2 vsT3.

### Left cardiac structural changes during pregnancy

The traditional echocardiographic parameters of the participants are demonstrated in Tables 2, 3. The LAD, LVMI, and ESV during the second and the third trimesters are increased compared to the first trimester and the control group. In the third trimester, RWT increased compared to the second trimester. EDV was higher in the second and third trimesters than in the control group. IVSd was the thickest during the third trimester. LVIDd and LVIDs were larger in the second and third trimesters compared to the control group. LVPWd in the third trimester was thicker than in the first trimester and the control group (Table 1).

**Table 2:** Echocardiographic cardiac structural parameters during pregnancy.

Variable	T1 (*n* = 149)	T2 (*n* = 99)	T3 (*n* = 258)	NPC (*n* = 172)	*P*
LAD (mm)	31.00 (28.00,33.00)^$^^,^^§^	32.00 (30.00,36.00)^†^	33.00 (30.00,35.00)*	30.00 (28.00,32.00)	<0.001
LVM (g)	111.7 (94.7,127.5)^$^^,^^§^	121.3 (104.1,140.7)^†^	127.5 (111.6,143.9)*	110.4 (97.2,127.1)	<0.001
LVMI (g/m^2^）	69.54 ± 11.62^$^^,^^§^	73.87 ± 12.14^†^	73.87 ± 11.05*	67.98 ± 11.71	<0.001
RWT	0.335 (0.303,0.375)	0.331 (0.301,0.371)^¶^	0.353 (0.318,0.393)	0.346 (0.311,0.372)	0.005
EDV (ml)	95.0 (83.7,104.5)^§^	101.9 (88.0,115.0)^†^	100.0 (92.0,112.2)*	90.9 (80.3,100.0)	<0.001
ESV (ml)	29.0 (24.2,33.1)^$^^,^^§^	32.0 (26.0,38.0)^†^	33.0 (28.9,38.1)*	28.0 (24.6,32.0)	<0.001
IVSd (mm)	8.00 (7.00,9.00)^§^	8.00 (8.00,9.00)^¶^	8.00 (8.00,9.00)*	8.00 (8.00,9.00)	<0.001
LVIDd (mm)	46.00 (43.00,48.00)^§^	47.00 (45.00,49.00)^†^	47.00 (45.00,49.00)*	45.00 (42.25.47.00)	<0.001
LVPWd (mm)	8.00 (7.00,8.00)^§^	8.00 (7.00,8.00)	8.00 (8.00,9.00)*	8.00 (7.00,8.00)	<0.001
LVIDs (mm)	28.00 (26.00,30.00)^§^	28.00 (27.0,31.00)^†^	29.00 (28.00,31.00)*	27.00 (26.00,29.00)	<0.001

Data are given as mean (standard deviation) or median (interquartile range). LAD, left atrial diameter; LVM, left ventricular mass; LVMI, left ventricular mass index; RWT, LV relative wall thickness; EDV, end-diastolic volume; ESV, end-systolic volume; IVSd, interventricular septal end-diastolic thickness; LVIDd, left ventricular end systolic diameter; LVPWd, left ventricular posterior wall thickness; LVIDs, left ventricular end diastolic diameter.

* *P* < 0.05, T3 vs. NPC.

^†^ *P* < 0.05, T2 vs. NPC.

^‡^ *P* < 0.05, T1 vs. NPC.

^$^ *P* < 0.05, T1 vs. T2.

^§^ *P* < 0.05,T1 vs. T3.

^¶^ *P* < 0.05, T2 vs. T3.

**Table 3:** Echocardiographic cardiac function parameters during pregnancy.

Variable	T1 (*n* = 149)	T2 (*n* = 99)	T3 (*n* = 258)	NPC (*n* = 172)	*P*
LVEF (%)	68.00 (65.00,71.00)^§^	68.00 (65.00,70.00)	66.00 (64.00,69.00)*	68.00 (65.00,71.00)	<0.001
E (cm/s)	100.00 (88.00,113.00)^‡^^,^^§^	96.00 (83.00,107.00)^¶^	83.00 (71.75,98.00)*	95.00 (83.00,105.75)	<0.001
A (cm/s)	61.00 (53.00,71.50)^$^^,^^§^	68.00 (58.00,77.00)^†^	65.00 (57.00,76.00)*	60.00 (54.00,68.00)	<0.001
E/A	1.60 (1.35,1.90)^$^^,^^§^	1.40 (1.20,1.70)^†^^,^^¶^	1.30 (1.15,1.50)*	1.50 (1.30,1.80)	<0.001
e’ septal (cm/s)	12.00 (11.00,13.45)^$^^,^^§^	11.00 (10.00,13.00)^¶^	9.00 (8.00,10.60)*	12.00 (10.55,13.00)	<0.001
Lateral e’ (cm/s)	17.00 (15.00,19.00)^§^	16.60 (15.00,18.00)^¶^	14.10 (12.00,16.15)*	16.60 (15.00,18.00)	<0.001
E/e’	6.80 (5.90,7.70)	6.70 (5.95,7.90)	7.00 (5.92,8.20)*	6.50 (5.70,7.50）	0.023
SV (ml)	69.88 (60.89,78.94)	74.58 (65.40,84.95)^†^	71.80 (63.73,81.85)*	66.12 (57.33,76.76)	<0.001
SI (ml/m²)	42.86 (37.91,48.23)	45.90 (39.59,51.13)^†^^,^^¶^	41.71 (36.09,47.15)	40.10 (35.40,46.10)	<0.001
LV-GLS (%)	−19.00 (−21.40, −16.70)^$^^,^^§^	−17.40 (−20.10, −15.30)^†^^,^^¶^	−16.35 (−17.93, −13.97)*	−19.10 (−20.80,−16.60)	<0.001
PA-TDI (ms)	114.19 (105.61,121.11)^§^	111.32 (107.27,121.11)^¶^	117.65 (108.45,128.03)	117.65 (107.27,124.57)	0.001

Data are given as mean (standard deviation) or median (interquartile range). LVEF, left ventricular ejection fraction; E, peak early diastolic transmitral valve velocity; A, peak late diastolic transmitral valve velocity; septal/lateral e’, peak early diastolic tissue Doppler velocity at septal/lateral mitral valve annulus; SV, stroke volume; SI, stroke index; LV-GLS, Left ventricular global longitudinal strain; PA-TDI, P-wave to A’ duration on tissue Doppler imaging.

* *P* < 0.05, T3 vs. NPC.

^†^ *P* < 0.05, T2 vs. NPC.

^‡^ *P* < 0.05, T1 vs. NPC.

^$^ *P* < 0.05, T1 vs. T2.

^§^ *P* < 0.05, T1 vs. T3.

^¶^ *P* < 0.05, T2 vs. T3.

### Left cardiac function changes during pregnancy

LVEF in the late pregnancy period was lower compared to early pregnancy or the nonpregnant group. The E wave in early pregnancy was higher than that in the control group, while in late pregnancy, it was lower than that in the control group. The A wave in mid and late pregnancy was higher than in early pregnancy and the control group. As gestational weeks increased, the E/A ratio gradually decreased. The lateral wall e' in late pregnancy was lower compared to that during early and mid-pregnancy, as well as the control group. As gestational weeks progressed, the interval e' gradually decreased. The E/e' ratio in late pregnancy was higher than in the control group. SV in mid and late pregnancy was higher than that in the control group. The SI in mid-pregnancy was higher than in late pregnancy and the control group.

With increasing gestational weeks, the absolute value of LV-GLS gradually decreased [T1 group, −19.00 (−21.40, −16.70); T2 group, −17.40 (−20.10, −15.30); T3 group, −16.35 (−17.93, −13.97); *P* < 0.001]. Furthermore, LV-GLS in mid and late pregnancy was worse compared to the control group. More importantly, PA-TDI in the third trimester was longer than that in the first [117.65 (108.45,128.03) ms vs. 114.19 (105.61,121.11) ms, *P* = 0.012] and second trimesters [117.65 (108.45,128.03) ms vs. 111.32 (107.27,121.11) ms, *P* = 0.010)] (Table 2). Multivariate regression analysis showed that gestational age was independently associated with LV-GLS (*b* = 0.096, *t* = 2.212, *P* = 0.027) and PA-TDI (*b* = 0.158, *t* = 2.449, *P* = 0.014). The results are shown in the Tables 4, 5.

**Table 4:** Multivariate analysis of LV-GLS in the healthy pregnancy group.

Variable	b	t	*P*	95% CI
Maternal age (years)	0.096	2.212	0.027	0.011–0.181
weight (kg)	−0.063	−1.292	0.197	−0.158–0.033
DBP (mmHg)	0.172	2.922	0.003	0.056–0.287
HR (bpm)	0.045	1.269	0.205	−0.024–0.113

**Table 5:** Multivariate analysis of PA-TDI in the healthy pregnancy group.

Variable	b	t	*P*	95% CI
Maternal age (years)	0.158	2.449	0.014	0.031–0.284
weight (kg)	0.195	2.716	0.006	0.054–0.336
DBP (mmHg)	−0.022	−0.25	0.802	−0.192–0.149
HR (bpm)	−0.097	−1.869	0.062	−0.199–0.005

### Reproducibility analysis

The intra-observer variability of PA-TDI measurements was assessed by the same sonographer (WXJ), 4 weeks apart from 20 randomly selected patients. The parameters were measured by another sonographer (CLL) to calculate inter-observer variability in PA-TDI. The results of the intra-observer and interobserver variability showed a strong agreement for PA-TDI, with the intraclass correlation coefficient (ICC) being 0.978 {[95% confidence interval (CI), 0.868–0.985]} and 0.965 (95% CI 0.826–0.969), respectively.

## Discussion

This study evaluated change in cardiac structure and function during pregnancy with echocardiography and showed subtle change in this special population, with a decline in both left ventricular systolic and diastolic function.

In our study, LAD begins to enlarge in mid-pregnancy, while LVIDd and LVIDs increase during late pregnancy, indicating that LAD may change earlier than LVIDd and LVIDs during pregnancy. Because the atrium has a thinner wall, it is more susceptible to change in blood volume and other factors, and the atrium frequently shows change before the ventricle (16). Moreover, we found thickening of the LV wall and an increase in LV mass in healthy pregnant women, markedly in late gestation. This was similar to the results of previous studies (17, 18). Previous research has shown that an increase of up to 50% in LVM and a 15%–25% increase in LV wall thickness during a healthy pregnancy (19). However, V.L. MEAH et al. (20) found no significant difference in LV wall thickness or mass between pregnant women and the control. This might be because the study only included pregnant women in the second trimester, and this change is only evident in late pregnancy.

We found that LVEF decreased in the late stage of pregnancy, but it remained within the normal range. This is consistent with the results of previous studies (17, 21). However, Orabona et al. (22) found that LVEF remained stable during singleton and twin pregnancies, and there was no significant difference between the two groups. The possible reason for the discrepancy is that LVEF has poor sensitivity in evaluating subclinical left ventricular systolic dysfunction. However, LV-GLS based on 2-dimensional speckle tracking has become the imaging modality of choice for detecting subtle cardiac dysfunction prior to LVEF reduction (12, 23). Previous studies have demonstrated that the absolute values of GLS, global radial strain (GRS), and global circumferential strain (GCS) were significantly reduced in women with hypertensive disorders of pregnancy (HDP) compared to the control group (24). There is also evidence that women with preeclampsia exhibit reduced GLS compared to those without (25, 26), with the impairment being more pronounced in the severe preeclampsia cases (26). These findings suggest that GLS can serve as a valuable non-invasive tool to detect early cardiac dysfunction in women with HDP. We found that the LV-GLS is reduced during the second and third trimester of healthy pregnancy, indicating that the trend of impaired GLS can already be observed during normal pregnancy, although it is less pronounced than that in the diseased conditions. Previous results indicate that although global longitudinal and circumferential strain of the LV decreases, radial strain increases (27). These compensatory adjustments allow the maternal heart to meet the hemodynamic requirements of pregnancy and labor while keeping overall LVEF performance within a normal range (27). In contrast, previous work by MEAH et al. (20) found that longitudinal strain and circumferential strain of the LV were increased in pregnant females compared to nonpregnant controls. They believe that the increased sympathetic activity and hormonal changes during pregnancy may play a role in the altered systolic function seen in healthy pregnancy, independently of any change in the overall hemodynamic load. The discrepancy may be explained by the assessment period in their research and the heterogeneity of the research population. The study was conducted within a narrow gestational window (22–26 weeks of gestation) and involved a small sample size: 18 non-pregnant women, 14 nulliparous pregnant women, and 13 primiparous postpartum women. We found that the absolute value of LV-GLS decreased as gestational age increases. Multivariate analysis also showed that gestational age is an independent factor affecting LV-GLS. This may indicate a gradual reduction in LV systolic function during pregnancy. The reason for this remains to be elucidated but may be related to maladaptation to hemodynamic change during pregnancy (21).

From the perspective of LV diastolic function, we found that E/A decreased in the mid-to-late pregnancy compared to early pregnancy, while E/e' did not show statistically significant change. This is similar to the results of a previous meta-analysis (3), which found that E/A gradually decreased after the second trimester and reached its lowest point between weeks 29 and 35 of pregnancy. It should be recognized that LV diastolic function evaluation requires the combination of various parameters. Each parameter alone has its limitation. The present result is unique in the application of PA-TDI to evaluate LV diastolic function. PA-TDI has been shown to provide a more comprehensive assessment of the extent of left atrial remodeling than other parameters (28). According to earlier research, PA-TDI was lengthened in the context of hypertension, atrial fibrillation, history of valvular illness, and higher BMI (29). The study by Leung et al. (14) confirmed that PA-TDI was a stronger predictor than left atrial strain in patients with paroxysmal atrial fibrillation. In multivariate analysis, we found that gestational age was independently related to PA-TDI. Evaluation of early change of LV diastolic function by PA-TDI. In conclusion, there is a trend of impaired LV diastolic function during pregnancy. Previous studies have also found a trend of decreased diastolic function during pregnancy (17, 21). The strength of this study lies in our use of multiple parameters to comprehensively assess the diastolic function in pregnant women. Our study also provides evidence that PA-TDI measurement is simple and convenient, providing a useful tool for the assessment of cardiac function in pregnant women.

This study conducted sequential observations of cardiac function in pregnant women during the first, second, and third trimesters to explore physiological change in healthy pregnancies. Besides, longitudinal observation of the postpartum cardiac function change in this cohort is underway to systematically evaluate the reversibility of pregnancy-associated cardiac functional change. Furthermore, we are prospectively investigating whether the restoration of cardiac function correlates with long-term clinical prognosis. The ongoing follow-up protocol includes monitoring for both adverse pregnancy outcomes—such as HDP, gestational diabetes mellitus (GDM), preterm birth, placental abruption, low birth weight, macrosomia, fetal growth restriction, and small-for-gestational-age infants—and longitudinal surveillance of cardiovascular events, including chronic hypertension, diabetes mellitus, coronary heart disease, and stroke.

### Clinical perspective

In recent years, cardiovascular disease in pregnancy has become the most common cause of maternal mortality, resulting in a heavy economic burden. Early detection of subclinical change in cardiac function and timely intervention is important for prevention and treatment of cardiovascular disease in pregnancy. LV-GLS and PA-TDI has been shown to be more sensitive than traditional echocardiographic parameters and are unaffected by volume load or measurement angle, allowing early detection of changes in cardiac function. In particular, PA-TDI has the advantage of being simple and convenient to measure, and if it is promoted for the assessment of cardiac function in pregnant women in the future, subclinical changes in cardiac function may be detected at an early stage, and timely interventions and treatments may be implemented to reduce the morbidity and mortality of maternal cardiovascular diseases in both the short and long term.

### Strengths and limitations

Most previous studies of cardiac function have focused on the middle and late stages of pregnancy, using widely varying indices, and the results remain controversial. In this study, we used a combination of conventional echocardiographic indices as well as PA-TDI and LV GLS, which are novel and sensitive indices to assess cardiac function during pregnancy. The drawback of this study was that it was a cross-sectional study, not a strictly speaking longitudinal study and despite recruitment procedures that attempted to match the groups as closely as possible, it was still impossible to completely exclude the impact of individual differences on study outcomes. Furthermore, this study currently lacks available long-term prognostic data on pregnant women and children for analysis. To more accurately assess change in cardiac function during pregnancy and to provide a foundation for the prevention and treatment of cardiovascular disease during pregnancy, future consecutive longitudinal studies are required for preconception, each trimester, and postpartum follow-up.

## Conclusion

The present study shows that there was some degree of cardiac remodeling and left cardiac function decline in the third trimester of pregnancy, but it is unknown whether this impairment will last or whether it will recover following delivery. Future study with larger sample and consecutive study is needed to investigate the longitudinal change in cardiac function during pregnancy.

## Data Availability

The raw data supporting the conclusions of this article will be made available by the authors, without undue reservation.
